# Mixed methods study of student participation and self-efficacy in remote asynchronous undergraduate physics laboratories: contributors, lurkers, and outsiders

**DOI:** 10.1186/s40594-023-00428-5

**Published:** 2023-05-17

**Authors:** Drew Rosen, Angela M. Kelly

**Affiliations:** 1 Institute for STEM Education, Stony Brook University, 092 Life Sciences, Stony Brook, NY 11794-5233 USA; 2 Department of Physics and Astronomy, University of Maine, 5709 Bennett Hall, Orono, ME 04469 USA; 3 Department of Physics and Astronomy, Stony Brook University, 092 Life Sciences, Stony Brook, NY 11794-5233 USA

**Keywords:** College/University Science, Laboratory science, Learning communities, Mixed methods, Physics, Remote learning

## Abstract

**Background:**

While laboratory practices have traditionally been conducted in-person, online asynchronous laboratory learning has been growing in popularity due to increased enrollments and the recent pandemic, creating opportunities for accessibility. In remote asynchronous learning environments, students have more autonomy to choose how they participate with other students in their laboratory classes. Communities of practice and self-efficacy may provide insights into why students are making their participation choices and how they are interacting with peers in asynchronous physics laboratory courses.

**Results:**

In this mixed methods, explanatory sequential study, students in an introductory physics remote asynchronous laboratory (*N* = 272) were surveyed about their social learning perceptions and their physics laboratory self-efficacy. Three groups of students were identified based upon their self-reported participation level of communication with peers in asynchronous courses: (1) *contributors*, who communicated with peers via instant messaging software and posted comments; (2) *lurkers*, who read discussions on instant messaging software without posting comments; and (3) *outsiders*, who neither read nor posted comments to peer discussions. Analysis of variance with post hoc Tukey tests showed significant differences in social learning perceptions among contributors, lurkers, and outsiders, with a large effect size, and differences between contributing and lurking students’ self-efficacy, with a small effect size. Qualitative findings from open-ended survey responses indicated contributors felt the structure of the learning environment, or their feeling of connectedness with other students, facilitated their desire to contribute. Many lurkers felt they could get what they needed through vicarious learning, and many expressed their lack of confidence to post relevant, accurate comments. Outsiders felt they did not have to, did not want to, or could not connect with other students.

**Conclusions:**

While the classroom laboratory traditionally requires all students to participate in the learning process through active socialization with other students, students in a remote asynchronous laboratory may still gain the benefits of participation through lurking. Instructors may consider lurking in an online or remote science laboratory as a legitimate form of participation and engagement.

## Introduction

There has been recent accelerating growth of remote asynchronous science laboratories in higher education, or hands-on laboratories conducted outside of a traditional laboratory classroom at home during hours determined by the student. This trend was catalyzed by the COVID-19 global pandemic and the growing enrollment demands placed on science departments. It is important to understand students’ social learning experiences because their social engagement in physics laboratory classes has been identified as a key facilitator of metacognition, self-efficacy, knowledge acquisition, and performance (American Association of Physics Teachers [AAPT], 2014). Exploration of the social learning experiences of online asynchronous laboratory students has been lacking in prior work, with researchers often opting to study performance outcomes (Merchant et al., 2014; Wei et al., 2019). Previous literature has indicated social experiences differ between students enrolled in asynchronous online vs. in-person physics laboratory classes (Fox et al., 2020; Rosen & Kelly, 2020, 2022). However, the reasons for these variations have not been well understood, particularly for students who take traditionally collaborative laboratory classes in remote, isolated learning environments. The present study expands upon previous studies to understand the nuanced nature of student social participation in an online asynchronous physics laboratory class informed by a social learning lens.

Participation and social interaction have often been considered important in the learning process. Students’ competence and success in academic classes are often related to their participation, which is frequently evaluated as part of their overall performance and further incentivizes participation (Kim et al., 2020). How professors define participation has varied and may include attendance, contributing to a classroom discussion, working in a group, and other forms of communication (Dancer & Kamvounias, 2005). Science laboratories provide a unique learning environment where students may engage with scientific phenomena and collaborate as part of a community of scientists (AAPT, 2014). Social participation in this process has the potential to improve students’ attitudes towards science and elicit cognitive growth, important outcomes of science teaching and learning (Hofstein & Lunetta, 2004), yet this process has not been studied extensively in remote asynchronous laboratory environments.

The demand for online laboratory instruction has grown dramatically over the last several years, partly because remote instruction offers a cost-effective solution for managing increased demand for laboratory instruction (Cooper & Ferreira, 2009). Remote asynchronous laboratory access also facilitates the participation of non-traditional students working or caring for others, those who cannot take a class at a specific time, or those with disabilities who do not have physical access to laboratory classes (Chen et al., 2010). Given the rapid proliferation of this type of asynchronous learning, it is useful to examine the nature and extent of social interactions in these settings. Researchers and policy makers have suggested that socially dynamic experiences in the physics laboratory facilitate scientific understandings, problem solving, data analysis, communication skills, and troubleshooting (AAPT, 2014; National Research Council, 2013; Zwickl et al., 2014), as well as students’ affective domains including self-efficacy, physics identity and self-concept, perceptions of physics relevance, and attitudes towards physics (Borish et al., 2022; Irving & Sayre, 2014; Nehmeh & Kelly, 2021; Rosen & Kelly, 2020, 2022). It is less clear how students socially interact when performing laboratory experiments remotely, and how the extent of interaction may be related to affective domains. Since science laboratories are typically collaborative in nature, it is important to understand how students may optimize their affective outcomes and conceptual learning when academic interactions occur remotely.

### Research questions

While the laboratory has been a longtime staple of science classes, online asynchronous laboratories, especially those using physical equipment, are an increasingly common offering by institutions to meet higher course demands and create access for more students. As new courses are developed, it is important they are designed with research-informed practices. Instructors new to online asynchronous learning may assume that minimal support and interactions are required for learners (Rovai, 2002a). There may also be an assumption that in-person students will inevitably engage with one another due in laboratory communities. However, students in online asynchronous communities may participate at different levels. This study will examine students’ participatory behavior in an online asynchronous physics laboratory by addressing the following questions:

1. To what extent do students in remote asynchronous physics laboratories engage in social interaction? What form(s) does this social interaction take?
2. What is the relationship between a students’ level of engagement within a community of practice and their self-efficacy?
3. How and why do students choose particular levels of social engagement with their communities of practice?

The present study is differentiated from prior work in its examination of students’ social interactions and self-efficacy in asynchronous remote laboratory classes, which have had received limited attention in prior research on students’ affective domains.

### Review of literature

While in-person classes have an advantage of physical proximity to facilitate student interactions, online classes require students to interact with one another in different ways. Remote asynchronous learning creates physical and psychological separation between students, often discouraging participation when compared to an in-person environment (Caspi & Blau, 2008; Lindsay et al., 2007). This requires new methods of defining and understanding participation (Ruthotto et al., 2020).

While students may use different strategies for socializing in remote classes than in-person students, this social process is often considered essential for learning (Hrastinski, 2009). However, the necessity of social interaction in science learning has not been consistently supported by the literature (Rosen & Kelly, 2022). For example, student learning and attitudes in collaborative groups often depend upon the balance between group goals and individual accountability (Pai et al., 2015). Also, research has shown that student outcomes in problem-based learning is more dependent upon personal engagement with the scientific phenomenon rather than the extent of social collaboration (Pease & Kuhn, 2011). This discrepancy in prior research suggests additional study is required to assess the impacts of socialization in science educational contexts, especially during the rapid shifts to remote instruction that occurred during the recent pandemic. Remote learning can allow for students to participate in classroom discussions with their peers without explicitly interacting with them.

Remote students engage through a virtual space, rather than through face-to-face interactions, to exchange information and provide or receive social support (Ridings & Gefen, 2004). While students in an in-person class who may not normally interact may be prompted by instructors (Dallimore et al., 2004), students in an online learning environment can choose to “lurk,” or read but not contribute to online discussions (Wenger et al., 2009). Lurking is a well-documented phenomenon within virtual discussion platforms (for example, Beaudoin, 2002; Honeychurch et al., 2017; Sun et al., 2014), and most people in large online communities are lurkers (Amichai-Hamburger et al., 2016; Wenger et al., 2002). Lurkers are by their nature invisible, making them difficult to track or study (Honeychurch et al., 2017). Lurking has often been perceived as a form of nonparticipation by educators, carrying with it the stigma of passive learners who are unengaged, often referring to them with negative terms such as “free-riders” (Bozkurt et al., 2020; Nonnecke et al., 2006). This can lead educators to misunderstand the nature of an online asynchronous learning environment (Edelmann, 2013), since students may learn both while posting and reading (Dennen, 2008; Xie, 2013). Consequently, lurking is not passive but rather an active form of participation and learning (Hrastinski, 2009; Nonnecke & Preece, 2003).

Motivational factors are particularly important in understanding participation in online asynchronous discussions (Hartnett, 2016; Xie et al., 2006), and researchers have identified many reasons why students choose to lurk. For example, self-efficacy, the belief in one’s capability of succeeding in specific tasks, is often a source of motivation (Bandura, 1997; Stephen et al., 2020), yet students with lower self-efficacy tend to be less likely to participate in online discussions (Kuo et al., 2014; Xie, 2013). Participation may be influenced by the level of domain knowledge (Cheung & Thadani, 2012; Nistor et al., 2014) or students’ perceived level of competence in the domain (Xie & Ke, 2011). In-person students who do not choose to interact because of low self-efficacy may be encouraged by instructors if they are observed struggling (Dallimore et al., 2004). Lurkers, because of their invisible nature, may not experience such an intervention prompted by spontaneous observation. Understanding the link between lurking behavior and physics laboratory self-efficacy in a remote laboratory class may reveal students’ reasons for doing so.

Time constraints may also influence participation (Amichai-Hamburger et al., 2016; Beaudoin, 2002). Students may feel it is sufficient just to read and reflect, which may be a form of vicarious learning or participation (Arnold & Paulus, 2010), especially if they see other students have already commented or asked questions similar to what they would have posted (Dennen, 2008). Students may prefer autonomous learning and choose not to engage with others (Moore et al., 2016), or they may not see the value in participating (Xie & Ke, 2011). If students feel there is a lack of postings or a lack of relevance in participating, or if there is too much information being posted leading to an overwhelming feeling, they may not participate (Cheung & Thadani, 2012). They also may not participate if they feel shy or feel as though they have nothing to offer (Nonnecke & Preece, 2003). If students do not feel comfortable with the technology used to engage with others, they may instead choose to lurk or not participate at all (Preece et al., 2004). Class size may also be a significant factor in students’ participation levels, with students in larger classes less likely to engage (Kim, 2013). Although many factors have been identified in previous studies to explain social interactions in online learning, few studies have examined these variables in undergraduate science laboratory classes, particularly in physics.

Peer interactions are the processes students use to engage in meaningful communications with one another, with or without the presence of their instructors (Vlachopoulos & Makri, 2019). This may include data or information sharing, helping or relying on others to solve problems, or the co-construction of knowledge. Online learning allows for technology to create an environment that allows students to engage with one another, potentially leading to optimal learning outcomes (Chen et al., 2010). Technology may also facilitate social presence, which is beneficial for students who are physically or psychologically separated (Rovai, 2007). This may lead to higher levels of perceived learning and satisfaction (Richardson et al., 2017).

### Theoretical framework

This study applies the community of practice framework and social cognitive theory related to self-efficacy to describe students’ social learning experiences in remote undergraduate physics laboratories and to provide insights into whether and how students chose to participate with their peers. Social interactions are relevant in laboratory learning since they often facilitate self-efficacy (AAPT, 2014); research in this area has been lacking since prior work has often focused on cognitive rather than affective domains (Brinson, 2015; Wei et al., 2019). These social constructs are discussed and operationalized in terms of communities of practice, the nature of peer interactions, and self-efficacy.

#### Communities of practice

The communities of practice framework, first posed by Lave and Wenger (1991) to understand the relationships between apprentices and experts, is here used to understand the structure and interactions of student online learning communities in a remote, undergraduate physics laboratory course. Wenger et al. (2011) defined a community of practice as:

...a learning partnership among people who find it useful to learn from and with each other about a particular domain. They use each other’s experience of practice as a learning resource. And they join forces in making sense of and addressing challenges they face individually or collectively (Wenger et al., 2011, p. 9).

Wenger himself expanded this framework into educational settings (Wenger, 1998), and communities of practice have since been applied to various educational settings including science laboratories (Irving & Sayre, 2014; Rosen & Kelly, 2022; Wheeler et al., 2017) and virtual learning communities (Ouyang & Scharber, 2017; Smith et al., 2017; Waycott et al., 2017; Wenger et al., 2009; Whitworth & Benson, 2016). Communities of practice are bound by their members’ *shared domain* (identity, common issues, and knowledge that brings the community together), *community* (members, relationships), and *practice* (the body of knowledge, tools, methods, and artifacts shared among members) (Wenger et al., 2002). Learning in this framework is socially constructed over time by negotiating meaning through various levels of participation (Wenger, 1998), which is often self-directed in online social educational platforms. However, instructors who have shifted to online platforms have reported challenges related to maintaining social presence and student engagement during this transition (Donham et al., 2022).

Central to engaging within a community of practice is the duality of participation and reification (Wenger, 1998). Participation is the “process of taking part and also the relations with others that reflect this process” including both “action and connection” (Wenger, 1998, p. 55). Participating in a community of practice is a reciprocal process, with members understanding that “making the community more valuable is to the benefit of everyone” (Wenger et al., 2002, p. 37). Reification represents the processes and products, both physical and conceptual, produced by community members. This can include creating, describing, using, interpreting, or recasting of artifacts (Wenger, 1998; Wenger et al., 2009). In online laboratories, students can combine participation and reification through sharing data or analysis and replying to or commenting on such information provided by other students (Wenger et al., 2009). Online learning often expands opportunities for participation and reification among students, potentially making it easier for a community to proliferate. It is through participation and reification that members create and negotiate the criteria for membership in the community of practice including the purpose of the community (joint enterprise), how members interact (mutual engagement), and the common resources shared between members (shared repertoire). These criteria also serve as a means of judging one’s own competence as a participant in the community (Wenger, 1998).

Communities of practice can offer various levels of involvement within the community, giving non-members pathways to more central participation as well as providing for members’ different desired levels of engagement or roles (Wenger, 1998). Newcomers to a community engage with members through legitimate peripheral participation, allowing them to work alongside central participants sharing knowledge, practices, activities, and identities (Lave & Wenger, 1991). It is in the periphery where people may be exposed to the practices of the community while reducing intensity, risk, or pressure to produce. Legitimacy for this kind of participation allows for newcomers to feel as though they could become full members (Wenger, 1998). For example, peripheral students in online laboratory classes might observe discussions to negotiate science misconceptions, connect theory to practice, and develop analytical approaches to problem solving.

While Lave and Wenger (1991) first presented legitimate peripheral participation as mutually exclusive with central membership, Wenger (1998) expanded upon this to allow for multiple roles and transitions. For example, rather than a singular, inward, trajectory from peripheral to central membership, members may choose to remain on the periphery to gain access to the community without intending to become full members. In virtual communities of practice, students may choose to engage in legitimate peripheral participation through lurking, or observing the interactions of others without engaging directly (Bozkurt et al., 2020; Lee et al., 2006). Lurkers often feel as though they are part of the larger learning community, experiencing a sense of membership and sharing values with other members (Honeychurch et al., 2017; Nonnecke & Preece, 2003). Reading other students’ posts, questions, and attempts to understand scientific principles is not a solitary, passive activity, but an active one that requires lurkers to interpret posts and the experiences of others through a social lens involving both individual and community perspectives (Edelmann, 2013).

#### Student–student interactions

Interactions serve as the foundation for building and sustaining online learning communities (Haythornthwaite & Andrews, 2011; Rovai, 2007). Reading the posts of others may serve as a source of motivation for participation and learning (Hartnett, 2016). Peer interactions in online learning environments have been shown to lead to higher learning satisfaction (Borish et al., 2022; Jung et al., 2002), more positive attitudes (Xie et al., 2006), increased performance (Picciano, 2002), and overall success (Menchaca & Bekele, 2008). Student interactions are often an important aspect of classroom laboratory practices (Hofstein & Lunetta, 2004), with the learning goal of facilitating students’ collaboration skills consistent with authentic science practices (Chinn & Malhotra, 2002). Peers may also negatively impact a desire to interact or participate in online learning communities if students feel as though others are not raising any interesting questions (Fung, 2004), there is a time delay between comments and responses (Cheung & Hew, 2004), or their comments are ignored or have not generated responses (Hew & Cheung, 2008).

Students do not necessarily need to contribute comments to engage in learning with their peers in remote learning environments. Learning may occur vicariously through observation of other students’ actions or behaviors (Bandura, 1997). Students may exert agency by placing themselves into environments and situations where vicarious learning may occur (Schunk & Usher, 2012). In an online learning environment, students may choose to lurk so they invisibly interact with their peers, feeling connected with others without risking failure. This may stem from a lack of confidence in their level of expertise (Wasko & Faraj, 2000), fear of criticism, or self-perceived risk of misleading others (Ardichvili et al., 2003). Students may also prefer no interaction with peers, allowing for independent learning; this has historically been considered a potential benefit of distance learning (Moore et al., 2016; Sun et al., 2014).

#### Self-efficacy

Self-efficacy has long been incorporated in sociocognitive frameworks providing a lens into behavioral motivation and cognitive and affective outcomes (Kelly, 2016; Schunk & Usher, 2012). A person’s self-efficacy, or belief in one’s ability to succeed at specific tasks, is based on previous academic and social experiences within one’s environment (Bandura, 1977, 1997). Self-efficacy has often been examined in educational settings because motivation derived from self-efficacy has been linked to cognitive and other affective outcomes (Schunk & Usher, 2012). Self-efficacy has been associated with academic achievement, diminished anxiety (Pajares, 1996), motivation (Schunk et al., 2014), self-regulation (Stephen et al., 2020), persistence through college (Holder, 2007), retention in science, technology, engineering, and mathematics (STEM) (Eccles & Wigfield, 2002), learner satisfaction (Kuo et al., 2014), and participation (Kuo et al., 2013).

Self-efficacy and students’ interactions with others and their environments are intertwined with one another. Self-efficacy may drive motivation to engage with others socially or through vicarious learning, which in turn may increase self-efficacy (Bandura, 1997). Interactions with other students may be more powerful sources of self-efficacy than interactions with instructors since students often identify classmates as role models for success (Schunk, 1987). These personal tendencies, environmental influences, and behavioral choices are dynamically related constructs that influence students’ affective domains and relationships with communities of practice (Bandura, 1989). In remote asynchronous learning focused on physics laboratory tasks, social interaction and self-efficacy constitute an insightful lens for examining students’ choices and identifying ways instructors might improve students’ affective outcomes.

## Methods

### Research design

The present study incorporated an explanatory sequential mixed methods design, in which quantitative data analysis was subsequently supported and expanded upon with qualitative data analysis (Ivankova et al., 2006). The quantitative data included Likert survey responses to measure students’ perspectives on their social interactions with peers and their self-efficacy in relation to physics laboratory tasks. Students were also asked to indicate the extent of their online communications with peers to understand their participation level and differentiate their participation from their views on their social interactions. Students indicated whether they participated in discussions with peers through explicitly contributing to the conversation or if they participated in the discussion by lurking. Some students were completely isolated and did not communicate with peers at all. This allowed the researchers to categorize students as contributors, lurkers, or outsiders, and to compare survey factors between groups. The classification of students into groups was guided by the theoretical framework of the study, in which participation in communities of practice may be differentiated by active contributors and those on the periphery (Lave & Wenger, 1991; Wenger, 1998). *Contributors* experienced community involvement through direct socialization and collaboration, distributing their expertise through public written posts and responses to the questions and statements of others; this involved both action and connection (Irving & Sayre, 2014; Wenger, 1998). *Lurkers* observed the written interactions of their peers without engaging with them directly, a form of peripheral participation (Bozkurt et al., 2020; Honeychurch et al., 2017; Lee et al., 2006). *Outsiders* chose not to participate in a community of practice. This group presented a unique perspective since students most often work in groups in traditional physics laboratory settings, yet the remote format did not require collaboration.

Qualitative data from open-ended survey questions were subsequently analyzed to determine why students chose particular levels of social participation. This explanatory lens allowed the researchers to address the complexity of the research questions by utilizing the qualitative findings to provide a more nuanced interpretation of the quantitative results (Subedi, 2016). The present study was approved by the Stony Brook University Institutional Review Board (#1316679).

### Study context

The context for this study was a large research STEM-focused university located in the Northeast United States, and data were collected once during the Spring 2021 academic semester. The university enrolled approximately 18,000 undergraduates, of whom 37% were White, 23% Asian, 12% Hispanic, 7% Black, 18% Non-resident Alien, and 3% Multiracial or Other. More than half of undergraduates (63%) majored in STEM or health sciences disciplines. The participants in the present study were primarily STEM majors enrolled in a single section of introductory calculus-based physics (*N* = 272), most of whom were first- or second-year students.

The laboratory course was the second of a two-semester sequence covering primarily electricity and magnetism. The learning goals included focus areas recommended by the AAPT, including constructing knowledge from personal observations, analyzing and visualizing real data, developing technical and practical laboratory skills, and presenting physics results with reasoned explanations (AAPT, 2014). Experiments were done asynchronously using a commercially available sensing device and associated software and kit that were purchased by students. They received written instructions on experimental procedures as well as embedded videos featuring an instructor performing the experiment. These student-performed experiments paralleled the typical in-person experiences, including electric field plotting and explorations of Ohm’s Law, Kirchoff’s Laws, RC and RLC circuits, magnetic forces and fields, Faraday’s law, Snell’s law, and diffraction. The devices had the capability to measure variables including current, resistance, voltage, capacitance, and electric and magnetic forces. These experiments were largely prescribed research questions with standardized procedures, although there were some opportunities for methodological variations and creativity. Students analyzed their data by using equations, reading and manipulating graphs, and calculating associated error. Students were evaluated based upon individually written laboratory reports, which counted towards 25% of their final grade in a four-credit physics course. Participation, that is engaging with peers in online forums, was not a graded element of the course and was not required.

Research has indicated students in online physics laboratory courses often struggle with unclear expectations, methodological questions, technological problems, and limited and/or infrequent immediate feedback from instructors and teaching assistants (Borish et al., 2022; Doucette et al., 2021; Rosen & Kelly, 2020, 2022). Consequently, students in the present study were invited by the instructors to participate in a voluntary *Slack* group, an online instant messaging program, at the start of the semester. This provided opportunities for them to engage with one another. In addition to a general chat channel within the group, separate channels within the *Slack* group were created for each laboratory experiment allowing for semi-organized, context dependent discussions. While teaching assistants were not always monitoring the chat channels, they were assigned specific office hours where they would monitor the group. There was a teaching assistant channel where students could specifically ask for help. Students could also independently form their own online communities using platforms such as *GroupMe* or *Discord* (alternative online instant messaging programs) outside of the purview of the course instructors, meet face-to-face, or engage with their teaching assistants via email exchange. These communication channels were optional and were created to facilitate discussion. Their personal choices of whether and how to participate were the focus of this study.

Due to the ongoing global pandemic during the 2020–2021 academic year, 78% of undergraduate students were commuters (in typical years, fewer than half of students were commuters). Consequently, more students were enrolled in online STEM lecture and laboratory experiences than was customary. However, this university had introduced online asynchronous introductory physics laboratories in 2016–2017 as an option for students, so the format had been in place for over four years at the time of this study.

### Survey instrument

A survey instrument was completed by 272 undergraduate physics laboratory students out of 506 possible students (54% response rate). The survey was sent to students electronically through Blackboard towards the end of the semester, and students were incentivized with homework credit to respond. This strategy was employed since the non-random, self-selected sample would have the same incentive as those who chose not to complete the survey, although the sample may have included more motivated students. This timing was intentional since previous research indicated that the second half of the semester is a critical period for examining student network development and how peer interactions might influence affective domains (Dou & Zwolak, 2019). The survey consisted of 24 five-point Likert scale statements relating to students’ views on their social interactions with their peers and their self-efficacy in completing physics laboratory tasks; the scale was 5 = strongly agree, 4 = somewhat agree, 3 = neither agree nor disagree, 2 = somewhat disagree, and 1 = strongly disagree. The questions were based upon prior research on students’ affective experiences in classroom communities of practice. The survey questions were selected and modified by the researchers from several previously developed instruments (see Table 1), and the resulting survey was validated for physics laboratory domain relevance and representativeness (Messick, 1989; Rosen & Kelly, 2020) by three experts in physics education. The 24-item survey was pilot tested with 223 students in a similar physics laboratory course during the prior year and demonstrated adequate reliability, however, previously published student outcomes did not involve an intervention (Rosen & Kelly, 2022). The overall reliability for the present study was also adequate (Cronbach’s* α* = 0.89), and the explained common variance of the instrument was 46.11%. An attention checking question was used to detect inattentive students by asking respondents to answer a question in a particular way (Curran, 2016). Students were asked, *“We use this statement to discard the survey of people who are not reading the questions. Please select somewhat agree (not strongly agree) for this question to preserve your answers,”* and 22 students who did not respond “correctly” were removed from the sample.

**Table 1:** Survey of undergraduate physics social learning perceptions and physics laboratory self-efficacy

Survey questions factor loading	
Factor 1: social learning perceptions (*α* = 0*.*91)	
1. I am able to depend on other students for help with my physics lab (Fraser et al., 1995)	0.74
2. When I have difficulty solving a physics laboratory problem, I like to discuss the problem with a peer (Mason & Singh, 2010)	0.56
3. Working with other students encourages and motivates me in this class (Fencl & Scheel, 2004)	0.43
4. I have little chance to get to know other students in my laboratory class (Fraser et al., 1995)	0.56
5. I feel that students in this course care about each other (Rovai, 2002b)	0.69
6. I feel connected to others in this course (Rovai, 2002b)	0.81
7. I feel that I can rely on others in this course (Rovai, 2002b)	0.88
8. I feel that members of this course depend on me (Rovai, 2002b)	0.54
9. I feel isolated in this course (Rovai, 2002b)	0.59
10. Members of my laboratory class help me (Fraser et al., 1995)	0.82
11. I find it easy to communicate with other students in my laboratory class (Rovai, 2002b)	0.73
12. I feel confident that others will support me (Rovai, 2002b)	0.70
Factor 2: physics laboratory self-efficacy (*α* = 0*.*90)	
13. I feel I could critique a laboratory report written by another student (Baldwin et al., 1999)	0.61
14. I am confident that I could read the procedures for an experiment and conduct the experiment on my own (Baldwin et al., 1999)	0.65
15. I am confident that I could use a scientific approach to solve an everyday problem outside of a classroom setting (Baldwin et al., 1999)	0.69
16. I am confident that I could analyze a set of data to answer a scientific question (i.e., look at the relationships between variables) (Barnett, 2011)	0.69
17. I am confident that I could tutor another student on how to write a lab report (Baldwin et al., 1999)	0.69
18. I feel comfortable expressing my opinions when others in my laboratory class disagree with me (Kost-Smith, 2011)	0.59
19. I feel I am capable of defending my physics ideas to my peers in my laboratory class (Kost-Smith, 2011)	0.74
20. Doing laboratory experiments and write-ups comes easy to me (Miller et al., 2015)	0.65
21. I feel I am capable of helping my classmates with physics in the laboratory (Miller et al., 2015)	0.76
22. I know how to explain what I do in the laboratory effectively (Miller et al., 2015)	0.74
23. I feel I can overcome any problems I encounter in an experiment (Kalender et al., 2020)	0.64
24. Before doing an experiment, I clearly understand the theory behind it (Dalgety et al., 2003)	0.53

Students were also asked to report their level of participation with others in their class, that is, the frequency they explicitly contributed to discussions with their peers. This allowed classification of students as contributors, lurkers, or outsiders, consistent with theoretical framing of levels of participation in communities of practice. Students selected the extent of their participation in a community of practice from the following characterizations: (1) *I did not communicate with other students and did not lurk or observe in any online forums or group chats*, (2) *I mostly observed or lurked in online forums, group chats, or other discussions but did not participate*, (3) *I participated or contributed infrequently (roughly once to three times a month),* (4) *I participated or contributed somewhat frequently (roughly once to three times a week),* (5) *I participated or contributed very frequently (roughly once to three times a day or more).* Students were also asked to provide their rationale for contributing, lurking, or not participating in online discussion forums.

In order to identify relevant constructs and revalidate the survey instrument, an exploratory factor analysis was performed with a direct oblimin rotation with Kaiser normalization (Field, 2013). Two factors emerged: (1) social learning perceptions (*α* = 0.91), and (2) physics laboratory self-efficacy (*α* = 0.90). The minimum primary factor loading was 0.43, which exceeded the suggested minimum of 0.4 (Stevens, 2012). Factor 1, identified as “social learning perceptions,” included 12 items. Questions from this factor related to a student’s sense of being part of a community of practice with their fellow classmates. These items collectively measured a student’s perceptions of peer interactions for learning physics in the laboratory, more specifically, fostering motivation, experiencing connectedness, growing confidence, and diminishing isolation. Factor 2, defined as “physics laboratory self-efficacy,” was also represented by 12 items. Questions related to students’ confidence in their capabilities to complete specific tasks in their physics laboratory courses. The short-term, task-specific nature of the self-efficacy items reflects students’ confidence in their ability to apply physics concepts in the laboratory, defend their ideas, perform experiments, analyze data, communicate results, transfer process skills to real world contexts, troubleshoot, help their peers, and evaluate the work of others. The survey items, sources, and factor loadings are represented in Table 1.

### Data analysis

#### Quantitative analysis

The explanatory sequential research design involved two phases, with quantitative data analysis performed before the qualitative analysis. First, descriptive statistics were generated and inferential tests were performed. Students were categorized into three mutually exclusive groups based upon their self-reported participation level within the virtual community of practice: (1) contributors (*n* = 110), (2) lurkers (*n* = 117), and (3) outsiders (*n* = 45). Students who posted any comments were categorized as contributors, those who merely observed were lurkers, and those who neither communicated nor observed were outsiders. These categories were modified from those used in prior research. For example, deWaard et al. (2011) used three labels for students in large enrollment online coursework: “memorably active participants,” “actively (contributing) participants,” and “potential lurkers.” Beaudoin (2002) labeled online students as having “high visibility” and “no-visibility.” In both studies, students were either socially engaged (leading or responding to discussions) or lurking (reading the posts of others without posting themselves). Additionally, these categories relate to levels of participation found in the communities of practice framework: (1) *outsiders* are students outside of the community, (2) *lurkers* are peripheral participants on the outside edge of the community, and (3) *contributors* are members inside of the community.

Analysis of variance (ANOVA) with post hoc Tukey tests was performed to measure between-group differences for each factor. The null hypothesis of no differences between contributor, lurker, and outsider groups with regard to social interaction and self-efficacy was tested. Students’ composite scores were calculated for each of the two factors, with higher scores indicating (1) more positive perceptions of student–student interactions in relation to completing laboratory tasks, and (2) more positive self-efficacy in performing these tasks. To meet the assumptions for ANOVA, Levene’s test indicated homogeneity of variance among groups for both Factor 1 (student–student social perspectives, *p* = 0.719) and Factor 2 (self-efficacy, *p* = 0.055). Based on a visual inspection of histograms and Q-Q plots, the data were found to be normally distributed. Data were independently collected from each student.

#### Qualitative analysis

In the second phase of the explanatory sequential research design, qualitative responses were compiled for the open-ended survey questions: “*Why did you use or choose this method? If you did not communicate or connect with other students in your class, please explain why you did not.*” The purpose of these questions was to elicit students’ reasoning for their preferred mode of communication, level of participation, and choice to participate.

The coding process was performed in three phases utilizing coding techniques identified by Saldaña (2009). The authors coded the responses independently for 40 students. During the first phase, *open coding*, they recorded first impressions of relevant phrases that explained students’ level of participation in the community of practice. This was followed by a discussion regarding the identified open codes and how they might be collapsed into fewer categories. Initially, there were 18 open codes for contributors, 20 for lurkers, and 11 for outsiders. After discussion, some open categories were combined into *axial codes*, where emergent categories were identified that grouped open codes into analytically similar constructs. Some open codes were eliminated at this point. The axial codes were based upon provisional codes previously cited in related literature and the theoretical framework (Saldaña, 2009), or they were identified through a grounded theory approach (Strauss & Corbin, 1998). After this phase, there were six axial codes for contributors, seven for lurkers, and three for outsiders. Once the designated axial categories were finalized, the researchers coded another 40 students to establish adequate interrater reliability (*k* = 0.74). The final phase involved *thematic coding*, where major explanatory constructs were identified and triangulated with quantitative results.

Students’ responses were analyzed both independently and collaboratively once they were identified as contributors, lurkers, or outsiders. The axial and thematic codes for each group are presented with response frequencies and representative comments. For each group, the percentages were calculated for the frequency of each axial code among responses. A response could be identified as more than one axial code.

## Results

### Quantitative results

A one-way analysis of variance (ANOVA) was performed to compare the calculated composite scores for each factor between contributors, lurkers, and outsiders; this method identified correlations among group membership and the constructs of social learning perceptions and physics laboratory self-efficacy. Inferential and descriptive statistics are presented in Table 2. For Factor 1, *social learning perceptions*, there were significant differences between groups (*F*(271) = 25.71, *p* < 0.001) with a large effect size (*η*_*p*_^2^ = 0.16). Post-hoc Tukey’s HSD indicated significant differences between outsiders and lurkers (*p* < 0.001), outsiders and contributors (*p* < 0.001), and lurkers and contributors (*p* = 0.005), with contributors responding the most positively followed by lurkers and then outsiders. For Factor 2, *physics laboratory self-efficacy*, there were significant differences between groups (*F*(271) = 3.14, *p* = 0.045) with a small effect size (*η*_*p*_^2^ = 0.02). Post-hoc Tukey’s HSD indicated significant differences between lurkers and contributors (*p* = 0.034) with contributors responding the most positively than lurkers. There were no significant differences between outsiders and lurkers or outsiders and contributors.

**Table 2:** Inferential and descriptive statistics of factor differences among outsiders, lurkers, and contributors

Factor	Group	*N*	Mean	SD	*F*	*p*	Effect size	95% Confidence interval	
							*(η*_*p*_^*2*^*)*	Lower bound	Upper bound
1. Student–student social learning perspectives	Contributors	110	38.70	9.01	25.71	< 0.001	0.16	37.00	40.40
	Lurkers	117	34.95	9.06				33.29	36.61
	Outsiders	45	27.36	8.52				24.80	29.92
2. Physics laboratory self-efficacy	Contributors	110	45.45	9.96	3.14	0.045	0.02	44.09	46.82
	Lurkers	117	42.63	8.96				40.99	44.27
	Outsiders	45	43.80	9.96				40.81	46.79

### Qualitative findings

Students’ written responses providing the reasoning behind their optional participation level were interpreted through a social learning and self-efficacy lens to provide insights into their behaviors. Two major themes emerged from their responses. Responses related to (1) internal, personal factors, or (2) external, social, or environmental influences. These themes were consistent with Bandura’s *triadic reciprocity* model, in which individual, behavioral, and environmental factors are dynamically interrelated (Bandura, 1989). In the present study, students’ social behaviors, internal dispositions, and environmental conditions influenced each other in a bidirectional fashion. Some responses were coded into both categories.

Personal factors included how students viewed or evaluated their own capabilities, affective domains relating to students’ comfort levels or other feelings, or other internal motivational factors. Social/environmental influences included the logistical and mechanical aspects or structure of the online learning environment, including aspects related to the number or availability of other students or the usability of a particular online platform. Students may also have been influenced by their relationships with one another through direct interactions, allowing them to learn or receive feedback from others. They could also engage in vicarious learning by reading about what others posted.

#### Contributors

In terms of social and environmental factors, contributors overwhelmingly attributed their participation level to the infrastructure of the learning environment itself, including the existing social connections, the significant number of students available, and other benefits of the online platforms. Another frequently mentioned social factor was their interpersonal connections to other students through shared learning challenges, allowing them to learn from one another. A small number of students also felt that most of their problems could be solved on their own, only engaging with other students as a last resort. This indicated that posting frequency varied among members of this group. Several students also noted how their questions posted online would go unanswered by their classmates.

In terms of personal factors, some students recognized the benefits of reciprocal communication. By answering other students’ questions, a contributor could benefit in the future from other students wanting to reciprocate and answer their questions. Some students commented on how they posted infrequently because of a lack of self-efficacy out of fear of being judged. These code frequency percentages are reported in Table 3.

**Table 3:** Contributors’ reasons for level of participation in community of practice (n = 88)

Theme	Axial code	% of respondents
Personal	Reciprocity	9.09
	Limited posting because of lack of self-efficacy	3.41
	Infrastructure	76.13
Social/environmental	Interpersonal	36.36
	Last resort	7.95
	Limited posting because of unanswered questions	3.41

Contributors frequently reported it was easy, quick, accessible, and convenient to communicate, which they often attributed to the user-friendly infrastructure of the instant messaging platforms. They were able to connect to many classmates, and most reported they had formed or entered the physics laboratory community through previously existing networks or communities. Two students, quoted below, noted how this infrastructure was especially helpful during the COVID-19 pandemic:

*It allowed me to reach out to a larger group of students for help or to help fellow classmates of mine with any questions they had. Using GroupMe is a great way to stay in touch with peers when classes are online and when it’s difficult meeting up face to face due to the pandemic.*

*I feel like due to COVID it is hard to meet up with peers in person. So once I found out about GroupMe I felt more comfortable to see that other students and I are in the same boat if we have any problems. It was as helpful as if I had a question, I had other peers help me get to the answer.*

These students leveraged the instant messaging platform to expand and/or strengthen their community of peers, sharing information and collaboratively addressing common questions. The convenience and rapid communication seemed to be a valuable replacement for the face-to-face interactions that typically dominated STEM laboratory classes. Communicators experienced strong interpersonal connections with their peers, expressing how they could learn from other students facing similar issues, allowing them to receive reliable feedback and feel supported. One representative student expressed the interpersonal benefits of belonging to a *GroupMe* chat, along with the confidence in the accuracy of information disseminated by other students:

*There were many students in the GroupMe and it is pretty accurate. It's mostly people asking questions and others helping explain how to solve them or helping each other come to the solution. It's a very helpful and supportive GroupMe.*

Several contributors discussed the feeling of reciprocity, a desire to help others with understanding physics. They valued the ability to help others benefit from their actions. This altruistic tendency was often facilitated by the tangible benefits they had received from others on the platform in the shared domain:

*Whenever I asked questions in the class GroupMe, I would always get an answer from another student. If I feel that I need further help, I was able to personally message them. After I finished my lab reports, I made sure that I was also helping other students with questions they had on theirs because it helped me, as well.*

This mutually beneficial back-and-forth communication was a motivation for continued engagement in the online community of practice, consistent with prior research (Hartnett, 2016). However, a few students noted that some questions would go unanswered by their classmates, resulting in frustration and disappointment while limiting their desire to communicate. A few students would only communicate with their classmates as a last resort if their teaching assistants or other sources of information failed them. This suggests that although contributors may often have exhibited internally motivated social engagement, the actions of others influenced their posting behaviors and active involvement.

#### Lurkers

There were several factors that contributed to lurkers’ self-reported lack of active engagement with their peers. In terms of social and environmental factors, nearly half of these students expressed how lurking offered them an avenue to learn or interact with their classmates vicariously. Some others noted how, like the contributors, the nature of the learning environment contributed to their decision to lurk. They felt comfortable with the platforms and the social infrastructures available to them, however, weaker interpersonal relationships were often a significant factor in choosing to lurk. Several students reported they did not feel close to or trust their fellow classmates, so lurking was a more comfortable way to engage with the community. Lurkers also reported personal reasons for observing discussions without contributing to them. Some stated they lacked the self-efficacy that would otherwise drive them to contribute. Other students experienced timing issues, for example, missing the cluster of postings that addressed a physics topic close to a due date. Others felt it was unnecessary to communicate with other students, or simply did not want to do so. These code percentages are reported in Table 4.

**Table 4:** Lurkers’ reasons for level of participation in community of practice (n = 94)

Theme	Axial code	Percentage of respondents
Social/environmental	Vicariously learning	46.81
	Infrastructure	20.21
	Interpersonal	11.70
Personal	Timing	5.32
	Lacking self-efficacy	17.02
	Unnecessary	10.64
	Did not want to	5.32

Lurking students frequently reported they could receive the benefits of receiving help from peers vicariously without actively engaging. Watching other students post questions and answers was often sufficient, since students were informed of challenges others had experienced. This improved their metacognitive awareness, since their own learning challenges resonated with those of their peers, and they could witness how students resolved challenges. One representative student explained her tendency to lurk as a means to anticipate potential issues:

*I mostly find the labs to be straightforward but having access to the GroupMe is helpful when students [do] labs ahead of time and "warn" us of problems to be aware of. Last semester I was not a part of a GroupMe, and while I see little changes in my grades, I believe seeing what others think about weekly labs has been helpful in my overall understanding.*

Another student noted how vicarious learning fit well with his personality, since he did not feel comfortable engaging with discussions with large groups of students. Lurking provided knowledge of fundamental concepts, motivation for tackling problems, and a sense of normative understanding in relation to his peers:

*I am not really the type of person to be active in large group chats, but I thought that lurking in these forums gave me a lot of inspiration and sometimes important or necessary knowledge for conducting the experiments.*

Other students noted certain benefits to infrastructure of the learning environment such as how easy or accessible it was, how comfortable it made them feel, or the large number of students in their learning community. Some students had issues with peer interactions, noting a lack of trust in information coming from other students, with one student noting, *“Students are not always a reliable source of help in this class. They could be just as confused as you are.”* Some students also commented on a lack of closeness to others, with inactivity or pauses on the social platform often leading to overall diminished communication.

The most significant personal factor related to lurkers’ chosen behaviors was self-efficacy. Many students lacked the confidence in their own capabilities or how much they knew, with some noting that they were shy or scared to post. There was also a concern for spreading misinformation, as this student explained: *“I don’t like expressing my own opinions because I am not sure if I am correct. I don’t want to be the cause for another student to fail if I am wrong.”* Some were also afraid of being judged, as one student expressed: *“I was scared I would be judged based off of my questions so I used it to see if any other students were having the same issues.”* Other students reported they either felt that it was not necessary to contribute or they simply did not have the desire. Students could get information from other sources such as their teaching assistants or the instructor-provided laboratory demonstrations themselves. One student said, *“The labs were clear enough to not need any external help. If I encountered any problem I was stuck on, it was generally already answered on the forums and I didn't need to ask.”* The lurkers were a unique group with regard to their passive engagement with the platform, however, they were still involved through observing the conversations of others, which often improved their metacognition, conceptual understanding, and normative self-assessment. Although this confirmed their membership in the community of practice (Honeychurch et al., 2017; Nonnecke & Preece, 2003), it was unclear the extent to which self-efficacy and lurking behavior were bidirectional, causal, or dependent upon individual traits.

#### Outsiders

Outsiders, who neither read nor posted communications with others, remained external to the learning communities for several reasons. Social and environmental factors included students feeling there were no students with whom to communicate, or it was unnecessary to do so. While communication was facilitated between students through an instructor-generated virtual learning community, not all outsiders were aware of its existence. Close to half of the students reported how communicating was unnecessary for their success while, in terms of personal factors, some others simply did not want to. These coded frequency percentages are reported in Table 5.

**Table 5:** Outsiders’ reasons for lack of participation in community of practice (n = 30)

Theme	Axial code	Percentage of respondents
Social/environmental	No people to communicate with	43.33
	Unnecessary	40.00
Personal	Did not want to	23.33

Many outsiders did not feel the need to communicate with others since they were confident in their own understanding and did not recognize any benefits from engaging with the community. Some students elaborated on this point by stating, *“Everything was explained clearly so I did not need extra help from my peers,”* and *“I was able to do all the experiments on my own and I never really needed help nor ran into any problems I couldn't figure out myself.”* However, some outsiders did not recognize the benefits of communication, which was not a requirement of the course, until they reached a point when they were struggling with the class. This student ultimately regretted her choice to be an outsider:

*I did not feel the need to communicate or connect with other students as this was a purely online class although looking back on it now, I wish I did seeing how much I am struggling in this class right now.*

Other students did not want to work with others because they were uncomfortable with the online nature of the course, preferring instead to work alone rather than engage in remote communication. Other students expressed their personal belief that communication was both unnecessary and uncomfortable: *“There were no group assignments and I don't enjoy working online with others as it feels awkward and more difficult than in real life.”* Many outsiders also felt that there was no one else with whom to connect. This was especially detrimental for this first-year student:

*I wasn't really sure who is in my class and I’m really new to the system so I am still at an introductory stage of my college life. I hope that I can slowly communicate with my peers more frequently.*

Although many outsiders were clear in their personal preference for working in solitude, it was evident that some of these students would have benefitted from more structured online discussions.

**Fig. 1:**
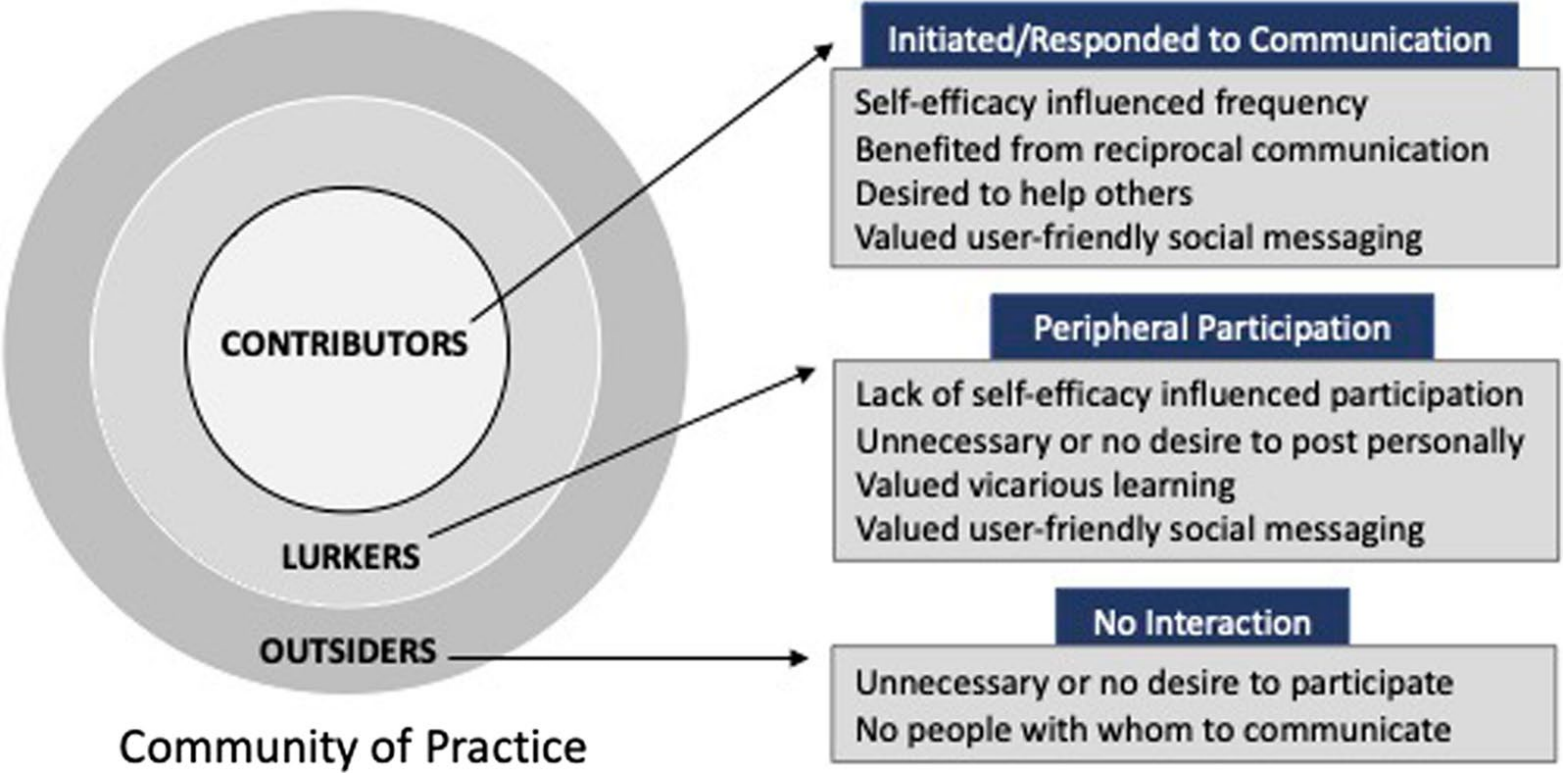
Internal and external factors influencing participation in community of practice

In summary, contributors, lurkers, and outsiders differed in their motivations for choosing to participate, the extent of their participation, their personal value of socialization, and their general self-efficacy. The qualitative findings for contributors, lurkers, and outsiders are summarized in Fig. 1.

## Discussion

This study identified differences in students’ perceptions of their interactions with classmates in an online asynchronous physics laboratory class, as well as differences in physics laboratory self-efficacy related to their participatory level within the community of practice structure. The social cognitive framework based on communities of practice and self-efficacy provided insights or potential explanations for these differences, which involved dynamic interactions among individual characteristics, environmental influences, and social behaviors (Bandura, 1989; Devi et al., 2017). While different levels of participation are a natural part of communities of practice, and previous work has examined them in online learning environments, they have not been well studied in the laboratory setting. Because of the unique challenges posed by teaching the science laboratory online, with the typically collaborative nature of laboratory work, it is important to understand students’ self-directed social learning experiences when they are taking laboratory courses online, physically separated from their classmates.

### Participation in a virtual community of practice

Different participatory roles are an important aspect of communities of practice, allowing for students to select how they wish to engage in a shared domain (Farnsworth et al., 2016; Wenger et al., 2002). In this study, students reported their participation levels, which were utilized to contextualize survey responses about their social learning perspectives. This construct demonstrated differences with a large effect size among groups. Contributors saw themselves as active participants within their communities and responded most positively to questions about their social learning perceptions. Lurkers saw themselves as legitimate members of their learning community, although they were clearly differentiated from their contributing peers, placing themselves on the periphery of the community. Outsiders viewed themselves having little or nothing to do with their peers and their choice not to communicate with peers reflected this view.

These significant quantitative differences among the three groups were further explained by students’ written responses. Contributors’ responses indicated the supportive nature and mutual benefits often found in a community of practice (Rosen & Kelly, 2022; Wenger et al., 2002). Students could engage in both participation and reification processes so they could learn from one another about physics principles and laboratory skills (Smith et al., 2017; Wenger et al., 2009). Some students explicitly noted the importance of reciprocity in their choice to contribute, an altruistic behavior often found in functioning communities of practice (Hou, 2015; Wenger et al., 2002). Lurkers most often cited the ability to learn vicariously as the reason for their lurking behavior, something also found in communities of practice (Sun et al., 2014), yet often not recognized or valued. By positioning themselves on the periphery, students may have benefitted from the virtual community of practice without becoming full-fledged contributors (Honeychurch et al., 2017). Outsiders mostly chose to exclude themselves from the community of practice, since many were confident in their performance and did not see value in participation. However, some were outsiders not by choice, but by circumstance. While instructors created a *Slack* channel to connect students, not every student was aware of its existence. Steps should be taken in future courses to ensure every student has access to their peers so that students’ participation choices are more intentional. This is particularly important when the choice to participate in group discussions is self-directed without external incentives from instructors.

Based on this differentiation of student roles, with lurkers and contributors working together in the same community structure, these students may be characterized as working within functioning virtual communities of practice at the periphery and core, respectively (Honeychurch et al., 2017). It is imperative that instructors recognize the potential value of students’ self-selecting participatory roles. For example, lurking may make the community more efficient, since there was a lack of redundancy when lurkers saw their questions were already addressed. Students also recognized the value of vicarious learning that was possible in a lurking role. Instructors who want all students to contribute to online discussions, especially in large virtual communities of practice, may inadvertently diminish the community’s value due to information overload (Gunaratne et al., 2020).

The roles that students assume in laboratories may influence how they perceive themselves. Working in a virtual community of practice may offer opportunities for complete role autonomy as well as the anonymity that may not be possible in an in-person course, including peripheral participation (Wenger et al., 1998). In-person laboratories typically require students to actively engage with one another to experience the benefits of the laboratory community of practice, which may include data sharing, problem solving, and task related troubleshooting. Lurking in a virtual community of practice may offer a low-risk role where students can learn vicariously by observing data, other information, or reification shared within the community, giving students a way to measure their own competence without having to make themselves visible to the community. The results and findings suggest that lurkers are active community members through interpreting the posts and learning challenges of others (Bozkurt et al., 2020; Edelmann, 2013), however, these students still had less positive views of their social interactions.

### Physics laboratory self-efficacy

Significant differences in students’ physics laboratory self-efficacy were found between lurkers and contributors, with contributors exhibiting higher self-efficacy, though the effect size was small, warranting further study. While contributors’ self-efficacy level was likely related to their centrality in the community of practice (Dou et al., 2016), a causal direction is unclear. Self-efficacy can be cyclic in nature creating a positive feedback cycle with sources of self-efficacy such as peer interactions or a community driven learning environment (Adams et al., 2020; Kelly, 2016). Students may have had higher self-efficacy to begin with, leading to their choice to contribute. It could also be that through interactions with classmates in a virtual community of practice, students experienced more validating experiences, leading to an increase in self-efficacy. While the results of this study do not establish causation, cycles like these are possibly occurring to a varying degree with different baselines for each student.

Lurkers’ lower self-efficacy was likely related to why they chose to lurk. Asking questions in an online chat room is an act of seeking feedback. Rather than risk, as some students put it, “looking stupid,” many lurking students would rather remain silent leaving their questions unasked. Students have reported a reluctance to ask for help to avoid social embarrassment (Kelly, 2016). For those students considering active verbal contribution, lurking offers a “safe” way for students to participate, allowing them to feel like members of the community until they feel comfortable enough to move out of the periphery towards becoming contributing members (Honeychurch et al., 2017; Nonnecke & Preece, 2003). Lurkers may also feel less comfortable engaging with their classmates because they are unfamiliar with an online learning environment or even the specific communication platform. This could negatively affect their self-efficacy.

Outsiders were not significantly different from lurkers or contributors in terms of self-efficacy. Their self-efficacy and desire for independent learning may have been a contributing factor to their choice to not participate or engage with other students (Moore et al., 2016; Sun et al., 2014). The autonomous learning behavior of outsiders may be accounted for by self-regulatory behavior, which self-efficacy can influence (Gatz et al., 2018; Honicke & Broadbent, 2016; Stephen et al., 2020). Self-regulation may be valuable in online learning environments where students have more responsibilities for their own independent learning (Bradley et al., 2017; Kuo et al., 2014). Outsiders may also feel as though they do not need to interact with other students because they get everything they need from interactions with their instructors and the online course content, two other potential sources of information or feedback.

### Limitations

This study had several limitations. The data were collected from a single university and thus may not be fully representative of the general population of undergraduate STEM students in the U.S. Participation in this survey was non-randomized and optional so the students who did participate were self-selected. Despite the opportunity for extra credit for participation, students’ responses may not have been representative of the population studied. The level of participation of students and the survey responses were all self-reported, although part of the aim of this study was to gain insights from students themselves about their participation. Because students primarily interacted with one another through avenues not accessible to instructors, self-reported responses could not be validated with analytics or network analysis. Students were not differentiated based upon their respective teaching assistants; the quality and frequency of instructional differences may have influenced their level of participation, their reasons for doing so, and their self-efficacy. Cognitive outcomes such as physics comprehension related to laboratory tasks were not measured, although previous literature has linked them to both participation and self-efficacy (Schunk & Usher, 2012). This study reported data collected during a single point in time. Participation, role within a community of practice, and self-efficacy may all evolve over the course of one semester. This study was also conducted during the COVID-19 global pandemic. Students’ self-efficacy and engagement could have been affected by anxiety and other factors relating to the significant disruption in traditional STEM undergraduate laboratory experiences. Another potential limitation of remote asynchronous instruction was the lack of incentives for students to engage in group discussions on online platforms. Results and findings could have been skewed by students’ variations in academic self-regulation.

### Future work

Future work may consider correlations between shifts in position in community structure with changes in self-efficacy, with the eventual goal of understanding the causal mechanisms between participation and self-efficacy. This may be achieved through repeated measures of factors such as social learning perspectives and self-efficacy. The relationship between self-efficacy and social learning should also be explored. While previous work has shown a relationship between the two (Gatz et al., 2018; Rosen & Kelly, 2022), pre-/post-measurements or structural equation modeling may identify a causal pathway. These causal relationships may provide insights for future intervention design or institutional resource allocation. Levels of participation in social networks is another promising are of research on remote laboratory instruction; there may be differential outcomes dependent upon social interaction frequency (Sundstrom et al., 2022a). Randomized controlled trials with more intentional conditions for student involvement in social networks may provide more nuanced insights on the relationship between student participation and cognitive and affective outcomes.

Performance outcomes based upon grades or standardized concept inventories should also be utilized since they may be predicted by participation level within an online course community. This is particularly important since research on the benefits of laboratory work with respect to students’ overall physics learning has been inconclusive (Wieman & Holmes, 2015). Qualitative data elicited by student questionnaires or interviews may provide insights into students' participation level choices. Long term, cross-institutional studies beyond a single course and university may reveal how social learning within virtual communities of practice influences intentions, levels, and mechanisms for participation. STEM retention is also another important long-term factor that may be influenced by varying participation levels in communities of practice. Longitudinal studies on students’ academic and vocational outcomes could provide insights on the long-term impacts of participation in laboratory-based communities of practice.

Gender has historically been shown to be a significant factor in participation and self-efficacy in STEM courses (for example, Fisher et al., 2020; Grossman & Porche, 2014; Nehmeh & Kelly, 2021; Rosen & Kelly, 2020), as well as participation in remote courses when communicating through online chat (Nichols et al., 2022). Groups where women are not in the minority can positively impact performance (Sullivan et al., 2018) and recognition (Sundstrom et al., 2022b), which can positively affect both participation and self-efficacy. Because the laboratory groups studied were not intentionally designed by instructors, rather students chose how to form groups and interact with one another, gender may have played a role in both how students participated and physics laboratory self-efficacy. Further work should be done to explore gender as a potential factor in the choice to lurk.

### Implications and conclusions

The STEM classroom laboratory traditionally requires all students to contribute to the learning process, mirroring the scientific process of a research community (Latour & Woolgar, 2013). However, in a remote or online laboratory class where students participate and engage with one another in a virtual community of practice, students do not have to contribute to be a part of the learning community. As seen in the present study, most students in the virtual community of practice did not contribute verbally. This does not mean, however, that instructors must be content with the status quo. While it is evident that lurkers are legitimate community members, they demonstrated a lower self-efficacy than contributors, which is some cause for concern given the role of self-efficacy in affective and learning outcomes (Kelly, 2016; Schunk & Usher, 2012). Instructional changes should recognize the role and value of legitimate peripheral participation. Though some lurkers may remain satisfied with their vicarious experiences throughout the course, some have the potential to become contributors yet may be hindered by a lack of self-efficacy. Not every student has to become a full-fledged contributor, but all should feel they may do so.

Course design choices for a remote asynchronous science laboratory course should be intentional and informed by a social cognitive framework and learning situated in a virtual community of practice. The STEM laboratory community is bound by the shared disciplinary domain and laboratory practice (Glaze-Crampes, 2020; Wenger et al., 2002), and the transition to remote asynchronous learning might consider options for maximizing the benefits of social engagement, whether peripheral or central to the community. This could include technology-supported assignments that require collaboration, such as long-term design projects (Marra et al., 2016). Online platforms should be chosen based on students’ prior experiences or comfort levels so they may leverage self-efficacy in their virtual learning space; an unfamiliar or non-user-friendly platform may be a barrier to participation. Instructors should also recognize that students’ motivation, behavior, and self-efficacy may vary depending on prior academic experiences, physics comfort level, and engagement in a community of practice. This may require additional training for faculty and teaching assistants beyond acting as moderators or facilitators in online discussions. It may also be advantageous for larger online classes to be broken up into smaller groups. Previous work has suggested lurkers in large groups may become contributors in small group discussions, moving from the periphery towards the community center (Honeychurch et al., 2017). Smaller groups would also match the class size typically experienced by in-person laboratory students (20–30 students, as opposed to 500 + in the present study). However, more research on the cognitive and affective outcomes of social interaction in the physics laboratory is necessary to evaluate whether such interventions should be targeted to specific students who may be more at risk for failure with participation in communities of practice.

Instructors possess the positional authority to impact students’ choices to participate in online discussions, which may provide students with opportunities for success early on to bolster their confidence (Caskurlu et al., 2021; Schallert et al., 2015). Access to guidance from instructors can significantly impact a student’s enjoyment of the course (Borish et al., 2022). For laboratories, this could mean having synchronous, small-group meeting times in the beginning of the course to ease students’ transitions into remote laboratory experiences. Such communications might also provide formative feedback for instructors, who often struggle with community building and fostering persistent student engagement in online instruction (Donham et al., 2022). Instructors may also create opportunities for self-evaluations to strengthen self-regulation, improve self-efficacy, and bolster early success. This may be achieved by incentivizing students to share laboratory techniques, data, and experimental findings, allowing students to see others overcoming similar challenges, thus increasing their beliefs in their own capabilities.

The science laboratory remains a critical component of science classrooms regardless of style or delivery mechanism. Because the demand for online asynchronous laboratories is likely to continue to grow, it is important for instructors to understand that the student experience, particularly *how* students participate, is different from the traditional classroom laboratory experience. However, students may work and thrive within communities of practice in both settings. Over the course of a semester in the traditional laboratory, instructors may observe which students are struggling to become more central members and which students are thriving. This may be more difficult in virtual asynchronous community of practice where lurking students often remain invisible to their instructors. This creates the potential for some students to be left behind as the community evolves over time.

Although this study captured only a snapshot of the community or practice near the end of the semester, it was evident that some students assumed lurking or outsider roles unable to move inward because of a lack of resources or self-efficacy. Understanding how and why students participate in various levels or roles in a remote asynchronous learning community is paramount for ensuring every student may optimize their laboratory experiences, physics learning, and affective outcomes.

## Data Availability

The datasets used and/or analysed during the current study are available from the corresponding author on reasonable request.

## Abbreviations

STEM: Science, technology, engineering, and mathematics
ANOVA: Analysis of variance
